# Thyroxine hormones visualized by the cryo-EM structure of bovine thyroglobulin

**DOI:** 10.1107/S2059798321011244

**Published:** 2021-10-29

**Authors:** Dušan Turk, Gregor Gunčar

**Affiliations:** a Institut Jožef Stefan, Jamova 39, 1000 Ljubljana, Slovenia; b Faculty for Chemistry and Chemical Technology, University of Ljubljana, Večna pot 113, 1000 Ljubljana, Slovenia

**Keywords:** iodine, bovine thyroglobulin, electron cryo-microscopy

## Abstract

A study of natively iodinated bovine thyroglobulin demonstrates that structural details of biologically important chemical reactions can now be visualized by electron cryo-microscopy.

In vertebrates, iodine has a unique role. Its circulation regulates metabolism and many other fundamental processes, including neuronal tissue growth (Caravalho & Dupuy, 2017 ▸; Di Jeso & Arvan, 2016 ▸). The cycle begins with the intake of iodine from the bloodstream into thyroid follicles by the Na^+^/I^−^ symporter (Dai *et al.*, 1996 ▸), where tyrosine residues of thyroglobulin (TG) are iodinated for iodine storage and hormone production. The coupling of two iodinated tyrosine residues forms the thyroid hormones T4 and T3, also called thyroxines. Hence, this giant molecule, TG, is used for the synthesis of the tiny thyroid hormone molecules. TG is a dimeric molecule and is known to every biochemist as it is used as the high molecular weight standard (330 kDa) for molecular weight determination by SDS–PAGE, yet its first 3D structure was determined only recently (Coscia *et al.*, 2020 ▸). Although the human TG structures were 93% complete, the obtained density maps lacked the resolution that could enable insight into such structural details. Moreover, the material obtained either from thyroid glands of patients with goitre or recombinant TG expressed in HEK293T cells was not iodinated.

In the cryo-EM structure of bovine TG from natural sources presented in this issue of *Acta Cryst. D* (Kim *et al.*, 2021 ▸), we see for the first time the intermediate of thyroxine hormone formation, the precursor of T4, composed of two iodinated tyrosine residues, together with all iodine atoms resolved in the map (Fig. 1 ▸). The structure provides unambiguous insight into the coupling of two pairs of tyrosine residues: acceptor Tyr24 and donor Tyr149, and acceptor Tyr2575 and donor Tyr2542. Moreover, the absence of density beyond the CB atom of residues Tyr149 and Tyr2542 in well resolved parts of the density maps is strong structural evidence for their donor role in hormonogenic reaction. These findings corroborate previous results of biochemical studies such as Lamas and coworkers (Lamas *et al.*, 1989 ▸) and site-directed mutagenesis of human TG (Coscia *et al.*, 2020 ▸), which identified four rather than two hormonogenic sites, including the alternative pairing of Tyr24 with Tyr149 and Tyr234. The Tyr234 residue was not iodinated in the bovine TG structure. These differences may arise from differences between the bovine and human thyroids and may also reflect the availability of iodine under *in vivo* conditions and those made available under the *in vitro* conditions of iodination assays (Coscia *et al.*, 2020 ▸). Kim *et al.* did not observe the presence of mono-iodinated tyrosine residues in the bovine TG structure. Similarly, biochemical assays performed by Coscia *et al.* did not reveal the presence of T3, the active form of thyroxine hormone. Hence, direct synthesis of T3 still needs to be demonstrated. The bovine TG structure exposes another interesting issue potentially related to the regulation of the redox potential of thyroid follicles. TG structures contain numerous cysteine residues, many of which are present in the cysteine-rich thyroglobulin type domains. In the human TG structure, all cysteine residues are linked by disulfide bonds. Due to the limiting resolution of density maps, this conclusion was often only supported by their proximity. In contrast, the bovine TG structure contains cysteine residues not involved in disulfide bridges despite their proximity, as described by Kim *et al.*

Although TG has been extensively studied, its three-dimensional structure could not be determined for decades. Isolation of purified TG (Heidelberger & Palmer, 1933 ▸) enabled the determination of its molecular weight (Heidelberger & Svedberg, 1934 ▸). The first complete sequence of TG was deduced from cDNA of bovine TG and contained 2769 amino-acid residues (Mercken *et al.*, 1985 ▸). Yet, a successful determination of a 3D structure requires sufficient amounts of homogeneous material and technology that makes the determination possible. The material has to be stable enough to sustain treatment by crystallization conditions or during the preparation of grids. It is not customary to publish unsuccessful attempts, but we believe that determination of the bovine TG structure was at least as long-lasting as that of its human homologue. Indeed, we know from personal experience how difficult this project was to bring to fruition. The structure of human TG was the culmination of about two decades of long endeavour, the seeds of which were planted by the crystal structure of MHC class II-associated p41 invariant chain fragment bound to cathepsin L (Gunčar *et al.*, 1999 ▸). The p41 fragment revealed the fold of thyroglobulin type-1 repeats, containing six cysteine residues and the CWCV signature (Molina *et al.*, 1996 ▸). For a long time, we struggled with attempts to crystallize TG from natural sources. Porcine material appeared to behave the best and was the easiest to isolate in large amounts. However, we were unable to obtain material that was stable over longer periods of time or reproducible crystals diffracting beyond 15 Å at a beamline such as the microfocus beamline at ESRF. During one visit (by DT) to MRC LMB, Jan Loewe suggested tackling the structure with electron cryo-microscopy, which was becoming capable of resolving the structures of macromolecules in atomic detail. Indeed, we progressed from porcine to purchased and later recombinantly expressed human thyro­globulin (Coscia *et al.*, 2020 ▸). As Kim *et al.* stated, the bovine TG material at first also appeared unstable on the grids. They hypothesized that the instability was caused by the inter­actions at the solvent–air interface and that a small addition of detergent already successfully applied in other studies (Noble *et al.*, 2018 ▸; Chen *et al.*, 2019 ▸) would prevent sample de­naturation. Indeed, they observed that the addition of CHAPS (CMC of ∼0.49%; GoldBio) prior to vitrification of the samples on the grid significantly improved the micrographs and resulted in a data set of 2.6 Å resolution. In addition to visualizing the T4 structure on the backbone of its precursor, the structure of bovine TG by Kim *et al.* is a triumph of sample preparation and the maturation of electron cryo-microscopy. The structure demonstrates that structural details of bio­logically important chemical reactions can now be visualized by electron cryo-microscopy.

## Figures and Tables

**Figure 1:**
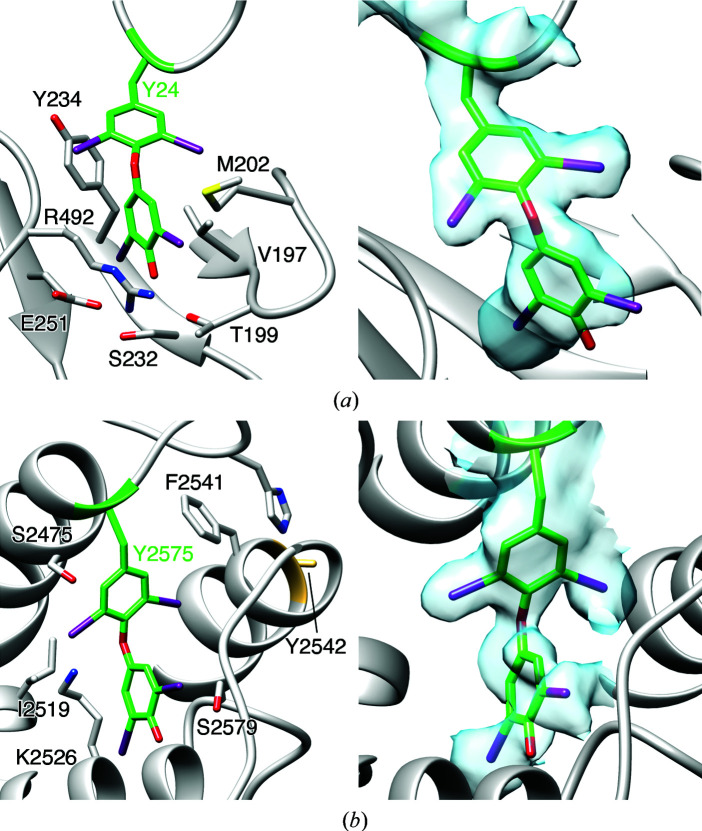
Iodotyrosines in bovine TG. Side chains and cryo-EM map density fit for thyroxinated side chains at Tyr24 (*a*) and Tyr2575 (*b*). Map contour level 0.032. Reproduced from Fig. 2 of Kim *et al.* (2021 ▸).

## References

[bb1] Carvalho, D. P. & Dupuy, C. (2017). *Mol. Cell. Endocrinol.* **458**, 6–15. 10.1016/j.mce.2017.01.038 28153798

[bb2] Chen, J., Noble, A. J., Kang, J. Y. & Darst, S. A. (2019). *J. Struct. Biol. X.* **1**, 100005. 10.1016/j.yjsbx.2019.100005 PMC7153306 32285040

[bb3] Coscia, F., Taler-Verčič, A., Chang, V. T., Sinn, L., O’Reilly, F. J., Izoré, T., Renko, M., Berger, I., Rappsilber, J., Turk, D. & Löwe, J. (2020). *Nature*, **578**, 627–630. 10.1038/s41586-020-1995-4 PMC7170718 32025030

[bb4] Dai, G., Levy, O. & Carrasco, N. (1996). *Nature*, **379**, 458–460. 10.1038/379458a0 8559252

[bb5] Di Jeso, B. & Arvan, P. (2016). *Endocr. Rev.* **37**, 2–36. 10.1210/er.2015-1090 PMC4740344 26595189

[bb6] Gunčar, G., Pungerčič, G., Klemenčič, I., Turk, V. & Turk, D. (1999). *EMBO J.* **18**, 793–803. 10.1093/emboj/18.4.793 PMC1171172 10022822

[bb8] Heidelberger, M. & Palmer, W. W. J. (1933). *J. Biol. Chem.* **101**, 433–439.

[bb7] Heidelberger, M. & Svedberg, T. (1934). *Science*, **80**, 414. 10.1126/science.80.2079.414 17842229

[bb9] Kim, K., Kopylov, M., Bobe, D., Kelley, K., Eng, E. T., Arvan, P. & Clarke, O. B. (2021). *Acta Cryst.* D**77**, 1451–1459. 10.1107/S2059798321010056 PMC8561740 34726172

[bb10] Lamas, L., Anderson, P. C., Fox, J. W. & Dunn, J. T. (1989). *J. Biol. Chem.* **264**, 13541–13545. 2760035

[bb11] Mercken, L., Simons, M.-J., Swillens, S., Massaer, M. & Vassart, G. (1985). *Nature*, **316**, 647–651. 10.1038/316647a0 3855243

[bb12] Molina, F., Bouanani, M., Pau, B. & Granier, C. (1996). *Eur. J. Biochem.* **240**, 125–133. 10.1111/j.1432-1033.1996.0125h.x 8797845

[bb13] Noble, A. J., Wei, H., Dandey, V. P., Zhang, Z., Tan, Y. Z., Potter, C. S. & Carragher, B. (2018). *Nat. Methods*, **15**, 793–795. 10.1038/s41592-018-0139-3 PMC6168394 30250056

